# Supplementary material for: Resolving the full spectrum of human genome variation using Linked-Reads
Source: Genome Res. 2019 Apr;29(4):635–45. doi: 10.1101/gr.234443.118 (PMC6442396; doi:10.1101/gr.234443.118)
Supplement: Supplemental Material [file supp_gr.234443.118_Supplemental_longranger-2.2.zip › longranger-2.2.tar.bz2!/lr2.2-public/lib/stan/cmdstan/stan/doc/api/html/rep__matrix_8hpp_source.html]

Stan: src/stan/math/rep\_matrix.hpp Source File


|  |  |
| --- | --- |
|  | Stan  2.5.0  probability, sampling & optimization |


- Main Page
- Related Pages
- Namespaces
- Classes
- Files

- File List
- File Members

All Classes Namespaces Files Functions Variables Typedefs Enumerations Enumerator Friends Macros Pages

- src
- stan
- math

rep\_matrix.hpp

Go to the documentation of this file.

1 #ifndef STAN\_\_MATH\_\_REP\_MATRIX\_HPP

2 #define STAN\_\_MATH\_\_REP\_MATRIX\_HPP

3

4 #include <boost/math/tools/promotion.hpp>

5 #include <stan/math/error\_handling/check\_nonnegative.hpp>

6 #include <stan/math/matrix/Eigen.hpp>

7

8 namespace stan {

9

10  namespace math {

11

12  template <typename T>

13  inline

14  Eigen::Matrix<typename boost::math::tools::promote\_args<T>::type,

15  Eigen::Dynamic,Eigen::Dynamic>

16  rep\_matrix(const T& x, int m, int n) {

17  check\_nonnegative("rep\_matrix(%1%)", m,"rows", (double\*)0);

18  check\_nonnegative("rep\_matrix(%1%)", n,"cols", (double\*)0);

19  return Eigen::Matrix<typename boost::math::tools::promote\_args<T>::type,

20  Eigen::Dynamic,Eigen::Dynamic>::Constant(m,n,x);

21  }

22

23  template <typename T>

24  inline Eigen::Matrix<T,Eigen::Dynamic,Eigen::Dynamic>

25  rep\_matrix(const Eigen::Matrix<T,Eigen::Dynamic,1>& v, int n) {

26  check\_nonnegative("rep\_matrix(%1%)", n,"rows", (double\*)0);

27  Eigen::Matrix<T,Eigen::Dynamic,Eigen::Dynamic> result(v.size(),n);

28  result.colwise() = v;

29  return result;

30  }

31

32  template <typename T>

33  inline Eigen::Matrix<T,Eigen::Dynamic,Eigen::Dynamic>

34  rep\_matrix(const Eigen::Matrix<T,1,Eigen::Dynamic>& rv, int m) {

35  check\_nonnegative("rep\_matrix(%1%)", m,"cols", (double\*)0);

36  Eigen::Matrix<T,Eigen::Dynamic,Eigen::Dynamic> result(m,rv.size());

37  result.rowwise() = rv;

38  return result;

39  }

40  }

41 }

42

43 #endif

check\_nonnegative.hpp

stan::math::rep\_matrix

Eigen::Matrix< typename boost::math::tools::promote\_args< T >::type, Eigen::Dynamic, Eigen::Dynamic > rep\_matrix(const T &x, int m, int n)

**Definition:** rep\_matrix.hpp:16

stan::math::check\_nonnegative

bool check\_nonnegative(const char \*function, const T\_y &y, const char \*name, T\_result \*result)

**Definition:** check\_nonnegative.hpp:52

Eigen.hpp


---

[ Stan Home Page ]

*© 2011–2014,
Stan Development Team.*
